# The 12 cm Masquerader: Struma Ovarii Disguised as a Benign Mucinous Cystadenoma (Ovarian-Adnexal Reporting and Data System 3) in a Young Woman

**DOI:** 10.7759/cureus.107017

**Published:** 2026-04-14

**Authors:** Rizwana Anjum, Mohammad Tariq Mahmood, Alaa Elghobashy

**Affiliations:** 1 Obstetrics and Gynaecology, American Hospital Dubai, Dubai, ARE; 2 Pathology, American Hospital Dubai, Dubai, ARE; 3 Gynaecologic Oncology, American Hospital Dubai, Dubai, ARE

**Keywords:** fertility-sparing surgery, monodermal teratoma, multiloculated ovarian cyst, o-rads 3, ovarian cystectomy, struma ovarii

## Abstract

Struma ovarii is a rare ovarian tumor that can clinically and radiologically resemble other benign adnexal masses, creating diagnostic challenges, particularly in reproductive-age women in whom fertility preservation is a priority. We report the case of a 30-year-old nulliparous woman who presented with a one-month history of pelvic fullness and a palpable lower abdominal mass. Imaging demonstrated a large multiloculated left ovarian cyst, interpreted as a benign lesion. Tumor markers were within normal limits, and the patient was clinically euthyroid. She underwent fertility-sparing left ovarian cystectomy with ovarian reconstruction, and the diagnosis of struma ovarii was established following surgical excision and histopathological examination. The postoperative course was uneventful. Struma ovarii should be considered in the differential diagnosis of complex ovarian masses, even when imaging and tumor markers suggest a benign etiology. Histopathological confirmation is essential for accurate diagnosis and management. In benign cases, fertility-sparing surgery is an effective treatment option.

## Introduction

Struma ovarii is a rare monodermal ovarian teratoma, accounting for approximately 0.5-1% of all ovarian tumors and 2-5% of ovarian teratomas [1,2]. It is characterized by the predominance of mature thyroid tissue and is usually benign. Thyrotoxicosis has been reported in a minority of patients, and malignant transformation is rare but documented [3,4].

Preoperative diagnosis of struma ovarii is challenging because its imaging appearance frequently overlaps with that of benign epithelial ovarian tumors, particularly mucinous and serous cystadenomas [5-7]. Even with modern imaging techniques, definitive preoperative identification remains difficult, especially in large multiloculated cystic lesions [6,7]. Typical imaging features may include multiloculated cystic architecture, variable internal echogenicity, and areas of solid components with vascularity; however, these findings are nonspecific and often mimic benign ovarian neoplasms.

Risk stratification tools such as the Ovarian-Adnexal Reporting and Data System (O-RADS) provide standardized assessment of adnexal masses; however, rare tumors such as struma ovarii may still be categorized as low-risk when classical malignant features are absent [8]. This diagnostic uncertainty complicates surgical planning, particularly in reproductive-age women desiring fertility preservation.

We report a case of a large benign struma ovarii that was misdiagnosed as a mucinous cystadenoma on imaging, highlighting diagnostic limitations, intraoperative decision-making regarding frozen section, and the potential future role of artificial intelligence (AI)-assisted imaging in improving preoperative characterization of adnexal masses [9].

## Case presentation

A 30-year-old gravida 0 para 0 healthy woman presented with a one-month history of lower-abdominal fullness and a palpable pelvic mass. She denied urinary or bowel symptoms and had regular menstrual cycles. She reported no personal or family history of thyroid disease.

Examination

The abdomen was soft and non-tender. A non-mobile, vaguely defined mass was palpable in the left lower abdomen.

Investigations

Tumor markers, including cancer antigen 125 (CA-125), Inhibin A/B, carcinoembryonic antigen, lactate dehydrogenase, and beta-human chorionic gonadotropin, were all within normal limits. Thyroid function tests were not performed as the patient was clinically euthyroid.

**Figure 1:**
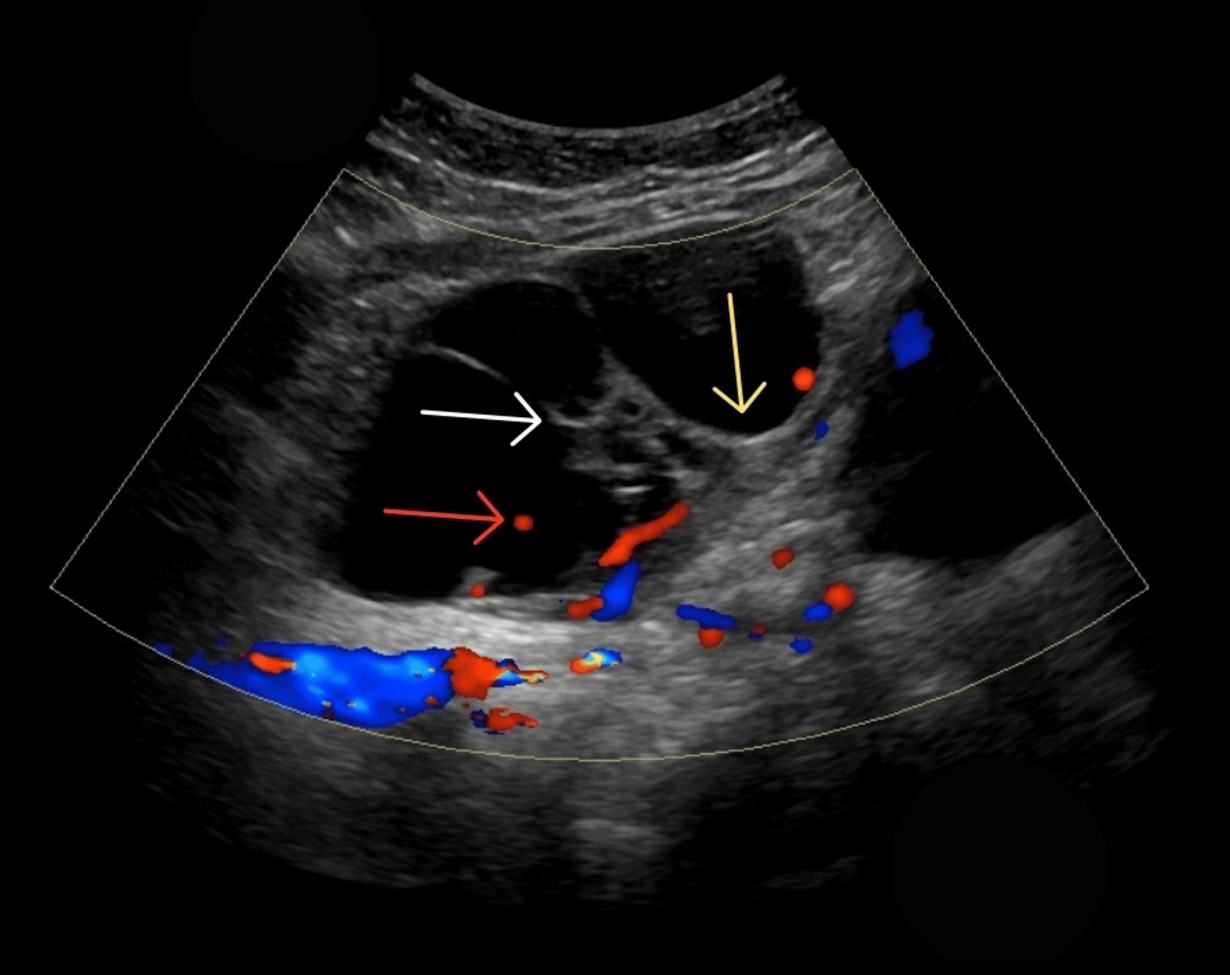
Pelvic ultrasound. Pelvic ultrasound demonstrating a multiloculated left ovarian cystic lesion with an internal solid mural nodule (white arrow) and thick septations (yellow arrow). Color Doppler reveals internal vascularity within the lesion (red arrow), raising suspicion for a neoplastic ovarian mass.

Pelvic ultrasound demonstrated a bulky left ovary with a multilocular cystic lesion measuring 12.2 × 11.5 × 7.0 cm. The lesion contained multiple septations and intracystic solid mural nodules with internal vascularity on color Doppler. A neoplastic etiology was suspected (Figure 1).

**Figure 2:**
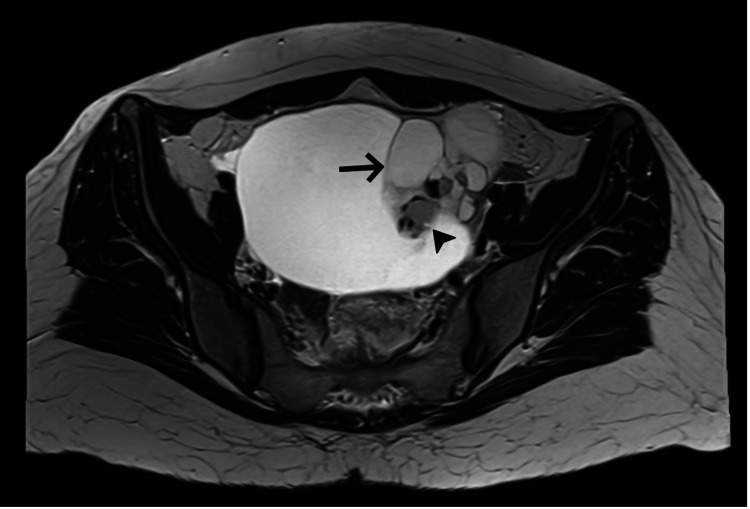
Axial T2-weighted MRI showing a benign-appearing multiloculated ovarian cyst mimicking an epithelial neoplasm. Axial pelvic MRI demonstrating a large multiloculated left adnexal cystic lesion. An internal septation (arrow) and a mural solid component (arrowhead) are identified, contributing to the preoperative suspicion of mucinous cystadenoma.

MRI of the pelvis confirmed a large (12 × 10 × 12.7 cm) multiloculated cyst with variable T1- and T2-weighted signal intensities and thin septa without significant mural nodular enhancement. The absence of diffusion restriction or significant solid enhancement supported a benign interpretation (O-RADS 3), reported as a mucinous cystadenoma (Figure 2).

Treatment

A fertility-sparing cystectomy was performed via laparotomy due to the lesion’s size and the risk of rupture. Peritoneal washings were collected for cytological evaluation.

Pathology

**Figure 3:**
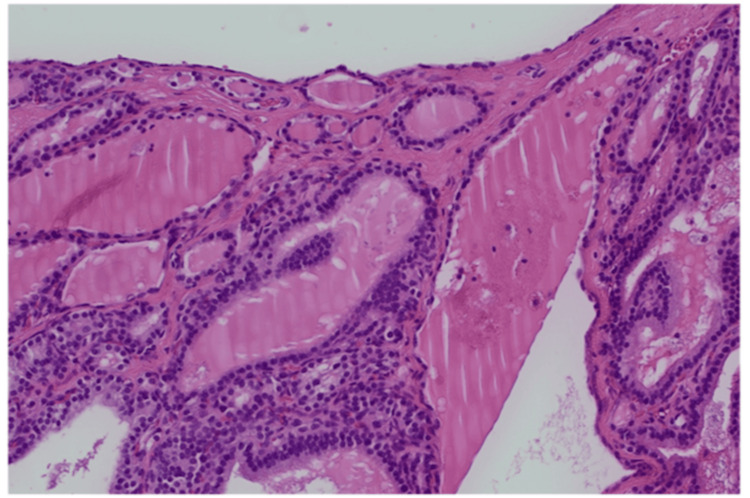
High-power histopathology (hematoxylin and eosin stain). High-power image showing thyroid follicles lined by uniform cuboidal to low-columnar epithelial cells with bland nuclear morphology, consistent with benign struma ovarii.

**Figure 4:**
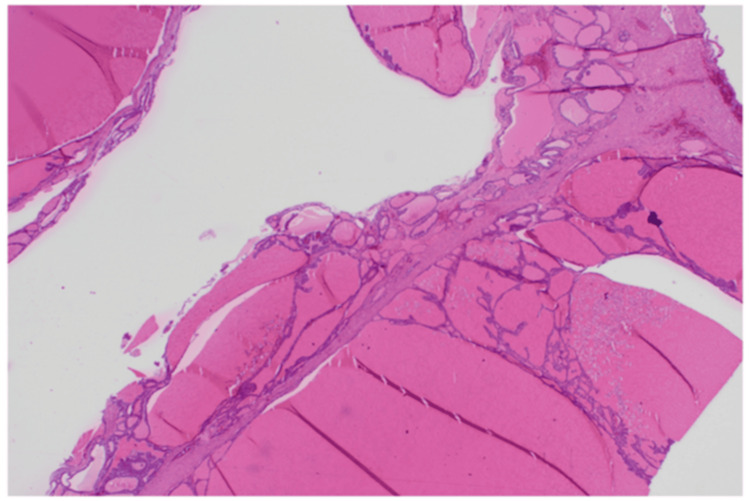
Low-power histopathology (hematoxylin and eosin stain). Low-power view showing abundant thyroid-type tissue composed of well-formed follicles filled with homogenous eosinophilic colloid, separated by delicate fibrous septa, features characteristic of struma ovarii.

Grossly, the specimen measured 9.5 × 5.5 × 4.5 cm and consisted of a multiloculated cyst with a smooth outer surface. Microscopically, >90% of the lesion was composed of thyroid follicles filled with colloid and lined by bland cuboidal to columnar epithelial cells (Figures 3, 4).

**Figure 5:**
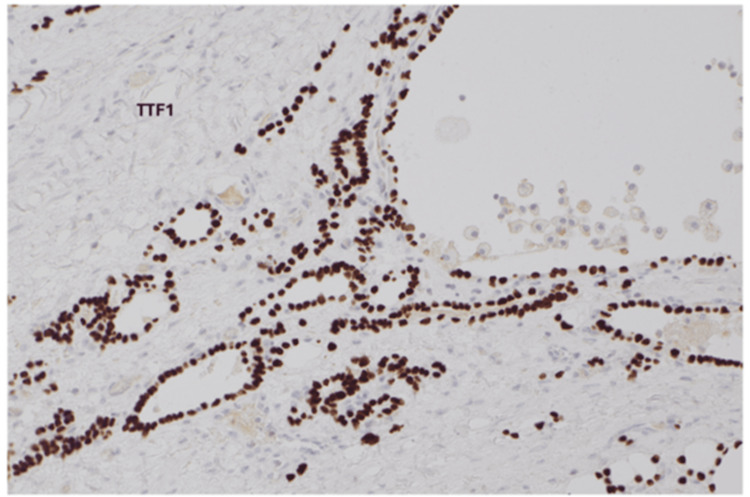
Immunohistochemistry (TTF-1). Immunohistochemical staining for thyroid transcription factor 1 (TTF-1) showing strong nuclear positivity in the follicular epithelial cells, confirming thyroid differentiation consistent with struma ovarii.

Immunohistochemistry demonstrated strong thyroid transcription factor-1 (TTF-1) positivity, confirming thyroid differentiation (Figure 5).

Cytological analysis was negative for malignant cells. The predominance of benign thyroid follicles without cytologic atypia, along with strong TTF-1 positivity, confirmed the diagnosis of benign struma ovarii and excluded malignant transformation. In the context of inconclusive imaging findings, histopathology played a decisive role in establishing the diagnosis and avoiding overtreatment. This case highlights the limitations of preoperative imaging in distinguishing rare monodermal teratomas from common epithelial ovarian neoplasms.

Postoperative recovery

The patient had an uncomplicated recovery and was discharged the next day in stable condition.

Follow-up

At the five-month follow-up, the patient was clinically well. Ultrasonography showed normal ovaries without residual or recurrent mass. CA-125, thyroid profile, and anti-Müllerian hormone levels were within normal limits, and no evidence of recurrence was identified.

## Discussion

This case highlights the principal diagnostic challenge of struma ovarii, which often presents with nonspecific clinical features and imaging characteristics indistinguishable from common benign epithelial ovarian tumors [5-7]. Many patients remain clinically euthyroid and demonstrate normal CA-125 levels, further reducing preoperative suspicion [3,4,10].

Ultrasound and MRI findings in struma ovarii are variable and frequently overlap with those of mucinous cystadenomas. Sonographic appearances may include multiloculated cystic masses with internal echoes or echogenic nodules, though these features may be subtle or obscured in large lesions [1,3,6]. MRI findings reflect heterogeneous tissue composition but are rarely diagnostic, contributing to misclassification within benign risk categories such as O-RADS 3, as seen in this case [7,8].

In this case, classification as O-RADS 3 was primarily based on the multiloculated cystic morphology, absence of significant solid enhancement, and lack of diffusion restriction, all of which favored a benign etiology. However, the presence of internal mural nodules and vascularity represents a diagnostic gray zone, underscoring the limitation of standardized risk stratification systems in rare tumors such as struma ovarii.

Emerging evidence suggests that AI-assisted imaging analysis may enhance preoperative classification of adnexal masses by integrating radiologic, biochemical, and clinical data. Recent studies indicate that AI-supported diagnostic models can improve diagnostic accuracy and reduce unnecessary radical surgery, supporting fertility-preserving approaches in appropriate patients [9].

Benign struma ovarii has an excellent prognosis following complete excision. Although most cases are benign, malignant struma ovarii represents a distinct clinical entity, and case series and systematic reviews have described the role of adjuvant radioactive iodine therapy in selected patients [11,12]. Malignant transformation is uncommon but necessitates individualized management strategies based on tumor behavior and extent of disease [13]. Definitive histopathological diagnosis remains essential for guiding follow-up and counseling.

Intraoperative frozen section can assist surgical decision-making; however, its accuracy is limited in large, cystic, or heterogeneous tumors due to sampling error and histologic complexity [13,14]. Misinterpretation of thyroid tissue may lead to unnecessary radical surgery. In patients with reassuring imaging and tumor markers who desire fertility preservation, selective omission of frozen section may be appropriate, as demonstrated in this case [14]. This case emphasizes that rare entities such as struma ovarii may be misclassified as low-risk on imaging, and careful integration of clinical, radiological, and intraoperative findings is essential to guide appropriate and fertility-preserving management.

Fertility preservation is a key consideration in the management of struma ovarii, particularly in young nulliparous women. Several recent reports support conservative surgical approaches, such as cystectomy or unilateral oophorectomy, in benign disease when adequate ovarian tissue can be preserved [14]. Careful patient selection, thorough intraoperative assessment, and definitive histopathological confirmation are essential to balance oncologic safety with reproductive outcomes.

## Conclusions

Struma ovarii remains a diagnostic masquerader that may be misclassified as a benign adnexal lesion on imaging, including O-RADS 3 categories. This case highlights the limitations of current preoperative assessment tools and reinforces the importance of histopathological confirmation. In carefully selected patients with reassuring clinical and radiologic features, fertility-sparing surgery without intraoperative frozen section may be considered. Emerging approaches, including AI-assisted imaging, may improve preoperative characterization of rare ovarian tumors. A limitation of this case is the absence of preoperative thyroid function testing, although the patient was clinically euthyroid.
